# Development and Optimization of Hybrid Polymeric Nanoparticles of Apigenin: Physicochemical Characterization, Antioxidant Activity and Cytotoxicity Evaluation

**DOI:** 10.3390/s22041364

**Published:** 2022-02-10

**Authors:** Ameeduzzafar Zafar, Nabil K. Alruwaili, Syed Sarim Imam, Omar Awad Alsaidan, Mohammed Muqtader Ahmed, Mohd Yasir, Musarrat Husain Warsi, Ali Alquraini, Mohammed M. Ghoneim, Sultan Alshehri

**Affiliations:** 1 Department of Pharmaceutics, College of Pharmacy, Jouf University, Sakaka 72341, Al-Jouf, Saudi Arabia; Nkalruwaili@ju.edu.sa (N.K.A.); osaidan@ju.edu.sa (O.A.A.); 2 Department of Pharmaceutics, College of Pharmacy, King Saud University, Riyadh 11451, Saudi Arabia; salshehri1@ksu.edu.sa; 3 Department of Pharmaceutics, College of Pharmacy, Prince Sattam Bin Abdulaziz University, Al-Kharj 11942, Saudi Arabia; mo.ahmed@psau.edu.sa; 4 Department of Pharmacy, College of Health Sciences, Arsi University, Asella 396, Ethiopia; mohdyasir31@gmail.com; 5 Department of Pharmaceutics and Industrial Pharmacy, College of Pharmacy, Taif University, P.O. Box 11099, Taif 21944, Saudi Arabia; mvarsi@tu.edu.sa; 6 Department of Pharmaceutical Chemistry, Faculty of Clinical Pharmacy, Al Baha University, Al Baha 65779, Saudi Arabia; aalquraini@bu.edu.sa; 7 Department of Pharmacy Practice, College of Pharmacy, Al-Maarefa University, Ad Diriyah 13713, Saudi Arabia; mghoneim@mcst.edu.sa

**Keywords:** breast cancer, hybrid nanoparticle, apigenin, cytotoxic activity, antimicrobial activity

## Abstract

Breast cancer is the most common cancer in females and ranked second after skin cancer. The use of natural compounds is a good alternative for the treatment of breast cancer with less toxicity than synthetic drugs. The aim of the present study is to develop and characterize hybrid Apigenin (AN) Nanoparticles (NPs) for oral delivery (AN-NPs). The hybrid AN-NPs were prepared by the self-assembly method using lecithin, chitosan and TPGS. Further, the NPs were optimized by Box-Behnken design (3-factor, 3-level). The hybrid NPs were evaluated for particle size (PS), entrapment efficiency (EE), zeta potential (ZP), and drug release. The optimized hybrid NPs (ON2), were further evaluated for solid state characterization, permeation, antioxidant, cytotoxicity and antimicrobial study. The formulation (ON2) exhibited small PS of 192.6 ± 4.2 nm, high EE 69.35 ± 1.1%, zeta potential of +36.54 mV, and sustained drug release (61.5 ± 2.5% in 24 h), as well as significantly (*p* < 0.05) enhanced drug permeation and antioxidant activity. The IC_50_ of pure AN was found to be significantly (*p* < 0.05) lower than the formulation (ON2). It also showed significantly greater (*p* < 0.05) antibacterial activity than pure AN against *Bacillus subtilis* and *Salmonella typhimurium*. From these findings, it revealed that a hybrid AN polymeric nanoparticle is a good carrier for the treatment of breast cancer.

## 1. Introduction

Flavonoids are the natural compounds found in plants that contain multiple phenolic units. The use of high flavonoid content reduces the chances of cancer [1]). The flavonoids directly control the cell differentiation and levels of the immune system [2]. Additionally, it can limit the activity of the mammalian target of rapamycin (mTOR), reduce the T-effector differentiation and stimulate T-regulatory cells [3]. Apigenin (AN) is a natural crystalline solid flavonoid, chemically it is a trihydroxy flavone (3-OH group). It is present in various natural sources like chamomile, artichokes, parsley and vine-spinach [4]. The various therapeutic activities like anticancer, antioxidant, antidiabetic, anti-inflammatory, anti-stress and antimicrobial were reported [5]. Various in vitro and in vivo studies reported the use of AN as an anticancer molecule by destroying cancer cells through activating cell cycle arrest, activation of an immune response, cell apoptosis and autophagy [6]. AN has poor water solubility, bioavailability and belongs to the BCS-II drug [7]. The researchers have tried to develop a novel nano-drug delivery system to increase the circulation time, drug release, cellular uptake and therapeutic potential [8]. Kashyap et al., reported nanoformulation-based delivery of various herbal bioactive compounds and evaluated them for in vivo anticancer activity as well as in a cell line study [9]. There are various approaches reported for increasing the solubility and therapeutic activity of poorly soluble drugs like cryptolepine-loaded solid lipid nanoparticles [10], black soybean nanoparticles [11], epicatechin and hydrogenated soybean-loaded hybrid nanoparticles [12], clofazimine and gefitinib-loaded polymeric nanoparticles [13,14]. The nanoparticle is one of the best ways to increase the solubility and therapeutic efficacy of low-soluble bioactive therapeutics. There are various polymers used for the development of nanoparticles, like chitosan (CS) [15], polylactic acid [16] poly(lactic-co-glycolic acid) [17] alginate [18], pectin [19], gelatin [20], and Eudragit^®^ [21] etc. Among them, chitosan (CS) is a natural bio-macromolecules polymer. CS is a polycationic and biologically safe polymer having many biopharmaceutical characteristics such as biodegradable, bio-adhesion and penetration enhancer. CS contains NH_2_ and OH functional groups and participated in the development of surface positive charge in acidic conditions so it can be used for surface modification [22].

Lecithin (LC) is a mixture of phospholipids and a bio-surfactant used for the stabilization of nanoparticles. LC has been employed for the development of liposomes [23], micelles [24], and nanoparticles [25]. d-α-tocopherol polyethylene glycol succinate (TPGS) is a derivative of vitamin E. It is biologically safe and approved by the FDA and widely used in formulations as well as acting as an emulsifier, solubilizer, stabilizer, and absorption enhancer. However, TPGS is extensively used as P-gp efflux transport inhibitor on the intestinal boundaries [26]. So, the incorporation of a therapeutic drug with TPGS and CS could improve its surface characteristics, P-gp inhibitory properties, improve stability and residence time.

In the present research work, we developed the Apigenin-(AN) loaded lecithin (LC)-chitosan (CS)-TPGS nanoparticles (AN-LC-CS-TPGS-NPs). The prepared nanoparticles evaluated were optimized by Box–Behnken design to select the optimum composition. The formulations were evaluated for particle characterization, encapsulation efficiency and solid-state characterization. Further, AN-LC-CS-TPGS-NPs were evaluated for in-vitro release, ex-vivo intestinal permeation, antimicrobial and cytotoxicity study on breast cancer cell line.

## 2. Materials

Apigenin and Chitosan were purchased from Beijing Mesochem Technology Co. Pvt. Ltd. (Beijing, China) and Spectrochem Pvt. Ltd., Mumbai, India. TPGS, lecithin, dialysis bag (MWCO 12–14 kDa), chloroform, acetic acid and antioxidant kits were procured from Sigma Aldrich (St. Louis, MO, USA). HPLC grade methanol, acetonitrile, and water were procured from SD-Fine Chemical (Mumbai, India).

### 2.1. Experimentals

#### 2.1.1. Optimization

AN-LC-CS-TPGS-NPs were optimized by Box–Behnken design using three independent variables at three levels (low, medium, high). LC (mg, A), CH (mg, B) and TPGS (%, C) were taken as independent variables and their effects were evaluated on particle size (nm, Y_1_), encapsulation efficiency (%, Y_2_) and drug release (%, Y_3_). The design showed fifteen formulations with three center points (*same composition) (Table 1). The data of each response were fitted into various experimental models (linear, 2nd order, quadratic) for the selection of a best fit model. The analysis of variance was calculated for the best fit model. The statistical regression analysis of each response was calculated to select the best fit model on the basis of regression co-efficient. 3D plots, contour plots and polynomial equations were generated to analyze the effect of independent variables over responses.

#### 2.1.2. Formulation of Apigenin Nanoparticles

**Figure 1:**
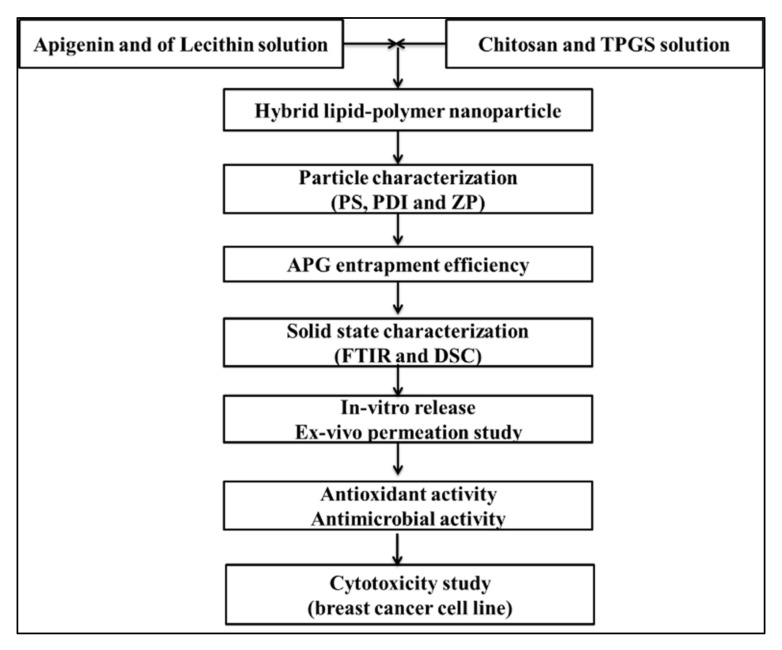
Flow chart of experimental design.

AN-LC-CS-TPGS-NPs were prepared by the molecular self-assembled method with slight modification [27], as shown in Figure 1. The AN-LC-CS-TPGS-NPs’ formulations were prepared by the given composition in Table 1. The weighed amount of AN (50 mg) and lecithin (80–180 mg) solution was prepared in a mixture of a methanol and chloroform blend (10 mL). Separately, CS solution (20–60 mg) was prepared in an aqueous acetic acid solution (1% acetic acid). The weighed quantity of TPGS (0.5–1%) was added to the CS solution. The CS/TPGS and LC/AN mixture solution was heated up to 60 °C and then LC/AN solution was added dropwise through a syringe into CS/TPGS solution under continuous stirring with a magnetic stirrer (10,000 rpm). The pH was adjusted to pH 4.5 using 0.1 *M* sodium hydroxide solution. The prepared AN-LC-CS-TPGS-NPs were centrifuged at 18,000 rpm for 30 min (cooling centrifuge Remi-24, Mumbai, India). The pellet was washed with Mili Q water and then lyophilized (Heto lyophilizer, Heto-Holten A/S, Denmark) to collect the final product.

### 2.2. Characterization

#### 2.2.1. Particle Size, PDI, Zeta Potential and Surface Morphology

The particle size, polydispersibility index (PDI) and surface charge (ZP) was analyzed by using the Malvern Zeta sizer (Ziss, Malvern, UK). The sample of AN-LC-CS-TPGS-NPs was diluted 100-fold with double distilled water and sonicated. The sample NPs were transferred to the cuvette for the analysis of particle size and PDI. The same prepared sample was transferred to a special cuvette having an electrode to measure the surface charge. The surface morphology of the prepared NPs was evaluated by transmission electron microscopy (Philips CM10, Holland). A drop of the AN-LC-CS-TPGS-NPs sample was placed over a copper-coated carbon grid and stained with one drop of phosphotungstic acid (2%). The excess liquid was removed and kept aside for staining. The stained sample was visualized under a high-resolution microscope to assess the morphology.

#### 2.2.2. Entrapment Efficiency

The entrapment of AG into NPs was determined by indirect method. The sample was filled into the tube and centrifuged at 10,000 rpm (Remi-24 Cooling centrifuge, India) for 30 min. The supernatant was separated and AN concentration was analyzed by UV-spectrophotometer at 268 nm. The EE (%) was calculated by using the formula:

(1) $$\mathrm{EE}\;\left(\%\right)\;=\frac{\mathrm{Total}\;\mathrm{amount}\;\mathrm{of}\;\mathrm{AN}-\mathrm{AN}\;\mathrm{in}\;\mathrm{supernatent}}{\mathrm{Total}\;\mathrm{amount}\;\mathrm{of}\;\mathrm{AN}}\times 100$$

#### 2.2.3. Fourier-Transform Infrared Spectroscopy (FTIR)

The FTIR analysis of the AN, TPGS, CS, LC, physical mixture and the optimized formulation (ON2) was done by using FTIR instrument (Perkin-Elmer, Waltham, MA, USA). Each sample was separately taken and the pellet was prepared with dried potassium bromide. The sample was scanned between 500–4000 cm^−1^ and the spectra were captured.

#### 2.2.4. Thermal Analysis

The thermal analysis of the AN, TPGS, CS, LC, physical mixture and the optimized formulation (ON2) was done by using a differential scanning calorimeter (Metler, Toledo, OH, USA). Each sample was placed in an empty DSC pan scanned between 25–400 °C under continuous flow of nitrogen for providing inert conditions.

#### 2.2.5. Drug Release Study

The release study of optimized AN-LC-CS-TPGS-NPs (ON2) was performed using a pretreated dialysis bag (MWCO-12000Da). One ml of AN-LC-CS-TPGS-NPs and pure AN (2 mg of AN) were filled into the dialysis bag and tied from both ends. The dialysis bag was immersed in a beaker containing 250 mL of phosphate buffer (pH 7.4 with 5% tween 80) under magnetic stirring (100 rpm). The temperature was maintained at 37 ± 0.5 °C during the whole study. At a defined interval, the released content (2 mL) was withdrawn and the same volume of release media was added to maintain the uniform study condition. The absorbance of collected samples was analyzed by UV-spectrophotometer at 268 nm. The concentration was calculated and the graph between time vs. percentage drug release was plotted. The release profile of optimized AN-LC-CS-TPGS-NPs (ON2) was fitted into different kinetic models and the graph was plotted using Microsoft Excel. The regression coefficient (R^2^) was analyzed to select the best fit model based on R^2^.

### 2.3. Ex-Vivo Permeation Study

The permeation study was done on an excised rat intestine. The intestine was collected after the immediate sacrifice of an albino Wistar rat and kept in chilled normal saline solution (0.9% NaCl). The intestine was cut into a size of 2–3 cm longitudinally. The intestine was washed, tied to one end and filled with 1 mL of optimized AN-LC-CS-TPGS-NPs (ON2) and pure AN dispersion (equivalent to 2 mg of AN). Both the ends of the intestine were tied tightly and dipped into ringer solution (pH 6.8). The continuous aeration (95% O_2_ and 5% CO_2_) was supplied by an aerator and maintained at 37 ± 0.5 °C. At a predetermined time (0.5, 1, 2, 3, 4 and 6 h) the released content (1 mL) was withdrawn and simultaneously replaced with fresh ringer solution. The sample was filtered through a membrane filter (0.25 µm) and the concentration of AN was estimated by the previously validated HPLC method (Cai et al., 2006). The permeation amount, flux and apparent permeation coefficient (APC) were calculated [28].

(2) $$\mathrm{APC}=\frac{\mathrm{flux}}{\mathrm{Area}\;\mathrm{of}\;\mathrm{permeation}\times \mathrm{amount}\;\mathrm{of}\;\mathrm{AN}\;\mathrm{in}\;\mathrm{formulation}}$$

### 2.4. In-Vitro Antioxidant Activity

DPPH method: The DPPH method was used for in-vitro antioxidant activity of pure AN and optimized AN-LC-CS-TPGS-NPs (ON2) as per the prescribed procedure with a slight modification [29]. The pure AN and the ON2 samples were prepared in methanol at a concentration of 1 mg/mL and the final concentration was diluted between 10–100 µg/mL. DPPH solution (0.02%) in methanol was prepared and 500 µL from each concentration of the AN and the ON2 was added into 125 µL of DPPH solution. The mixture was mixed by shaking and kept for 1 h in a dark place to complete the reaction. The color changed from violet to colorless, representing the antioxidant activity. The antioxidant activity was measured by a UV-spectrophotometer, the UV-Visible spectrophotometer at 571 nm. A comparative analysis of a control (ct) sample without DPPH analysis was also done and % scavenging activity was determined by the formula [29].

(3) $$\mathrm{Antioxidant}\;\mathrm{activity}=\frac{\mathrm{Absorbence}\;\mathrm{of}\;\mathrm{control}-\mathrm{Absorbance}\;\mathrm{of}\;\mathrm{test}}{\mathrm{Absorbence}\;\mathrm{of}\;\mathrm{control}}\times 100$$

ABTS radical scavenging method: The antioxidant potential of the pure AN and the ON2 was further assessed by the ABTS radical scavenging method using the previously reported method with slight modification [30]. The concentration 10–100 µg/mL range was prepared and 0.1 mL of each concentration of both samples was mixed with 0.9 mL of ABTS solution. The mixture was kept at room temperature in a water bath in dark conditions for 30 min. After completion of the reaction, the mixture was analyzed by UV-Visible spectrophotometer at 734 nm and compared with the control BHT solution. The study was performed in triplicate and antioxidant activity was calculated by the following equation [31].

(4) $$\mathrm{Scavenging}\;\mathrm{activity}=\frac{\mathrm{Absorbence}\;\mathrm{of}\;\mathrm{control}-\mathrm{Absorbance}\;\mathrm{of}\;\mathrm{test}}{\mathrm{Absorbence}\;\mathrm{of}\;\mathrm{control}}\times 100$$

### 2.5. Cytotoxicity Study

A cytotoxicity study of the pure AN and ON2 was done on a breast cancer cell line (MCF-7). The cell line was procured from American Type Culture Collection (ATCC, Manassas, VA, USA). The cell was grown on the DMEM media and pH was maintained to 7.4. The cell line was grown into a CO_2_ incubator (Galaxy^®^ 170R CO_2_ incubator, Eppendorf, Germany) by supplying 5% CO_2_ and 90% RH with penicillin (100 U/mL) and streptomycin (100 U/mL). The standard concentration of the AN and the ON2 (50, 100, 150, 200, 250 µM) was prepared in DMSO. Each concentration of the AN and the ON2 was placed into a 96-well plate containing 1 × 104 per well of MCF-7. MTT solution 0.05 mg/mL (MTT, Sigma, St. Louis, MO, USA) was added to each well and incubated for 2 h at 37 ± 0.5 °C under a continuous supply of CO_2_ (5%) and O_2_ (95%). Then, 100 µL of DMSO was added to each well to dissolve the formazan crystal. The resulting mixture was analyzed by a microplate reader (BioRad, Shinagawa-Ku, Tokyo, Japan) at 550 nm. Blank DMSO was also analyzed as a control. The inhibitory concentration (IC50) for the AN and the ON2 was also calculated and determined. The % cell inhibition was measured by the given equation.

(5) $$\mathrm{Cell}\;\mathrm{inhibition}\;\left(\%\right)\;=\frac{\mathrm{Absorbance}\;\mathrm{of}\;\mathrm{control}-\mathrm{Absorbance}\;\mathrm{of}\;\mathrm{test}}{\mathrm{Absorbance}\;\mathrm{of}\;\mathrm{control}}\times 100$$

### 2.6. Antimicrobial Study

The antimicrobial study of the AN and the optimized AN-LC-CS-TPGS-NPs (ON2) was done in Gram-positive (*Bacillus subtilis*) as well as Gram-negative (*Salmonella typhimurium*) strains. The study was performed by the cup plate method. The sterilized nutrient agar media was prepared and poured into a sterilized petriplate with microbial strain under aseptic conditions. The plate was kept in an aseptic area for complete solidification. The well (10 mm) was made by using a sterilized stainless-steel borer. The pure AN and the ON2 were filled into the well under aseptic conditions and stood for 15 min. The plate was placed into the incubator (Binder, Germany) at 37 ± 0.5 °C for 24 h. The zone of inhibition was measured by the graduated scale. The tetracycline solution was used as standard.

### 2.7. Statistical Analysis

The study was performed in triplicate and results shown as mean ± SD. The Student’s *t*-test was used for comparative study and *p* < 0.05 considered for significant effect.

## 3. Result and Discussion

### 3.1. Optimization

The formulation was optimized by three factor, three level Box–Behnken statistical design. The various formulations were prepared using independent variables: lecithin (lipid); chitosan (polymer); and TPGS (stabilizer). The variables were taken at low, medium and high levels as shown in Table 1. The selection of independent variables was done on the basis of preliminary study. The used composition showed significant effect on the particle size (Y_1_), encapsulation efficiency (Y_2_) and drug release (Y_3_). The low, medium and high level of each ingredient depicted variation in the results.

**Figure 2:**
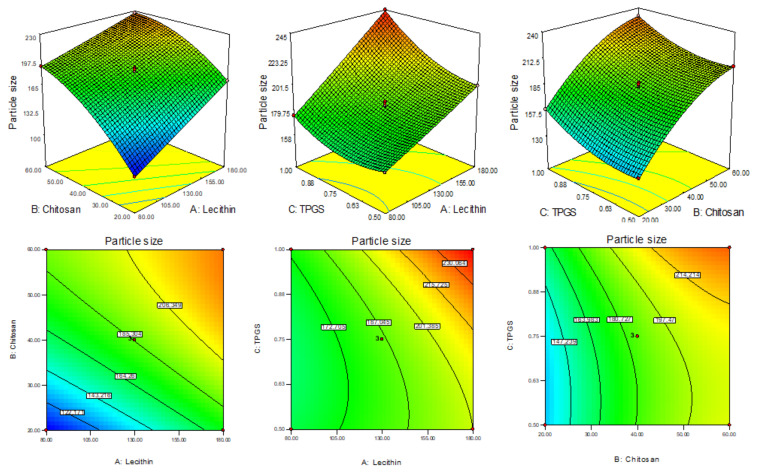
3D response surface plot showing the effect on independent variables on size (Y_1_).

**Figure 3:**
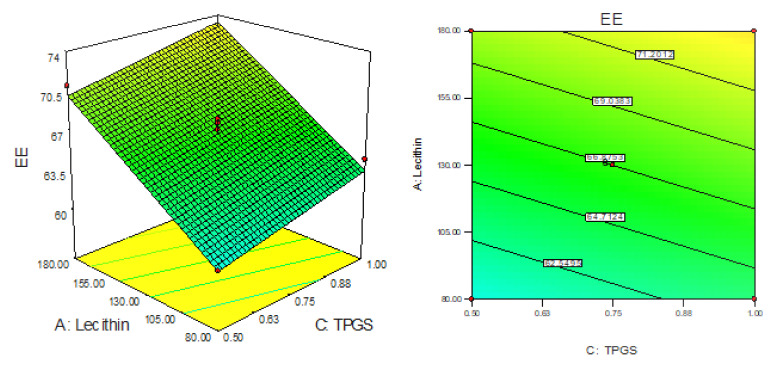
3D response surface plot showing the effect on independent variables on encapsulation efficiency (Y_2_).

**Figure 4:**
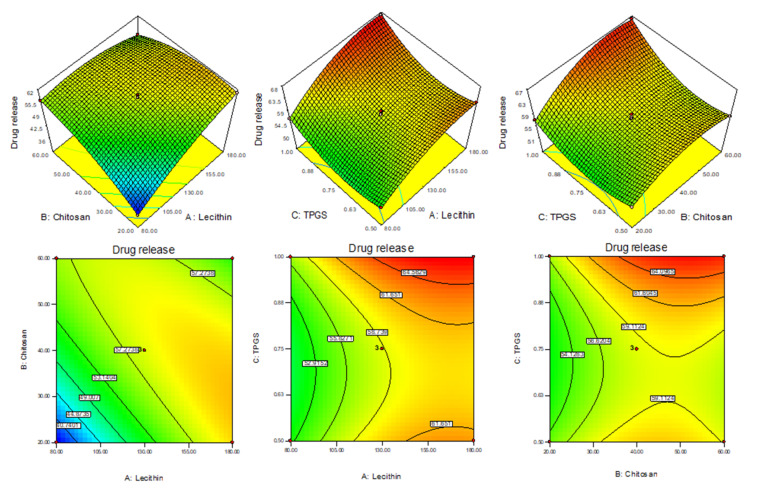
3D response surface plot showing the effect on independent variables on drug release (Y_3_).

The formulation responses were fitted into the different experimental models and the best fit was found to be quadratic for particle size (Y_1_), linear for EE (Y_2_) and quadratic for drug release (Y_3_). They have shown the maximum regression coefficient (R^2^). The analysis of variance of the best fit model for each response was evaluated and the values of Sum of Squares, F-Value and *p*-value (Prob > F) are given in Table 2. The polynomial equation and response surface plot (3D & contour plot) of all responses were generated (Figure 2, Figure 3 and Figure 4) defined the effect of formulation factors on response. The actual and predicted value graph was also plotted which explained the closeness between the actual and predicted value of responses.

### 3.2. Effect of Formulation Variable on Particle Size (Y_1_)

The PS of prepared AN-LC-CS-TPGS-NPs (F1-F15) was found in the range 101.28 nm (F1)–245 nm (F8) as shown in Table 1. The formulation (F1) prepared with LC (80 mg), CH (20 mg), and TPGS (0.75%) showed the lowest size and the maximum size shown by the formulation (F8) composition LC (180 mg), CH (40 mg), and TPGS (1%). The 3D response plot showed the effect of independent variables on the particle size (Figure 2). From the experimental design software, the polynomial equation for the particle size is given below:

Particle size (Y_1_) = 185.75 + 26.99A + 36.13B + 13.76C − 8.21AB + 7.71 AC − 0.47 BC + 0.56A^2^ − 13.83B^2^ + 9.61C^2^ (6)

The factors A, B and C are model-coded terms for LC, CS and TPGS. The positive and negative signs expressed synergistic and antagonistic responses. The model F-value was found to be 1475.44 points toward the model was well fitted. From the polynomial equation, it was found that as the concentration of LC (A), CS (B) and TPGS (C) increases the particle size also increases. The factors A, B, C, AB, AC, B^2^, C^2^ are the significant model term (*p* < 0.05) and the remaining factors BC and A^2^ are insignificant model terms (*p* > 0.05). The lack of fit is significant (*p* > 0.05) and it favors the fitted model. The adequate precession value >4 (145.02) showed the model was well fitted. The ANOVA of the fitted quadratic model was calculated and data are given in Table 2. On increasing the concentration of LC (A), CS (B) and TPGA (C), the particle size (Y_1_) was increased. With the increase in LC concentration (A), the particle size also increases due to enhancement in the viscosity of the solution. On increasing the CS concentration (B), the PS increases due to the increased viscosity and also provides resistance against stirring (agitation force) during the development of NPs [32]. On increasing the third-factor TPSG (C) concentration, the particle size also increased because TPGS covers the particle quickly and formed a high network as well as stabilizes the nanoparticle. The interaction between the negative charge of LC and the positive charge of CS promotes the increase in particle size. This finding agreed with previously published works [33,34]. The predicted R2 is 0.9980 is in close agreement with the adjusted R^2^ as shown in Table 3.

### 3.3. Effect of LC, CS and TPGS on Entrapment Efficiency (Y_2_)

The EE of AN-LC-CS-TPGS-NPs’ formulation (F1–F15) was found in the range of 52.65% (F1) to 79.54% (F4). The formulation (F1) prepared with LC (80 mg), CH (20 mg), and TPGS (0.75%) showed the lowest EE and the maximum EE was shown by the formulation (F8) composition LC (180 mg), CH (60 mg), and TPGS (0.75%). The 3D response plot showed the effect of independent variables on the encapsulation efficiency (Figure 3). The linear polynomial equation for entrapment EE was generated which gave the information of the effect on the formulation factors EE.

EE (Y_2_) = 66.87 + 4.90 + 8.17 + 1.58C (7)

The linear model was found to be well fitted for EE. The model term A, B and C are found to be significant. The F-value of the 164.68 exhibited model is significant and lack of fit found to be non-significant (*p* > 0.05). The predicted R^2^ of 0.9566 is in agreement with adjusted R^2^ (0.9722) as shown in Table 3. The LC (A), CS (B) and TPGS (C) show synergistic effects on EE. With the increase in the concentration of each independent variable the entrapment efficacy also increases. As the concentration of LC (A) and CS (B) increases, the EE of AN also increases due to the higher space available for entrapment of drugs during the interaction of LC (negative charge) and CS (positive charge). However, when the TPGS (C) concentration increases and the EE also increased due to the enhanced solubility of AN, this leads to accommodating more of the AN into the lecithin lipid core.

### 3.4. Effect of LC, CS and TPGS on Drug Release (Y_3_)

The drug release of all AN-LC-CS-TPGS-NPs’ formulations (F1 to F15) was found in the range of 36.47 (F1)–67.11% (F8) in 12 h. The formulation (F1) prepared with LC (80 mg), CH (20 mg) and TPGS (0.75%) showed the minimum release and the maximum release was shown by the formulation (F8) composition LC (180 mg), CH (60 mg) and TPGS (0.75%). The linear model was found to be well fitted for EE. The model term A, B and C are found to be significant. The LC (A), CS (B) and TPGS (C) show synergistic effects on EE. With the increase in the concentration of each independent variable the entrapment efficacy also increases. As the LC and CS concentration increases, the EE of AN into NPs increases due to the higher space available for drugs during the interaction of LC (negative charge) and CS (positive charge). However, TPGS concentration increases and the EE also increased due to the enhanced solubility of AN, leading to accommodating more of the AN into the lecithin lipid core. The 3D response plot showed the effect of independent variables on the drug release (Figure 4). The polynomial equation of quadratic model for drug release is given below which explains the effect of different factors on drug release.

Drug release (Y_3_) = 58.6 + 5.15A + 3.24B + 2.31C − 6.75AB + 0.21AC + 1.24BC − 3.18A^2^ − 3.663B^2^ − 4.30C^2^ (8)

In this equation the A, B, C, AB, BC, A^2^, B^2^, and C^2^ are significant model terms (*p* < 0.0001) and AC is an insignificant term (*p* = 0.45). The Model F-value of 287.73 expresses that the model is significant and well-fitted. The *p*-value of lack of fit is 0.78 (*p* > 0.05) and showed as non-significant. The adequate precision is >4 (72.81) indicating an adequate signal and good fit of the quadratic model. The predicted R^2^ is 0.9861 and it is in reasonable agreement with the adjusted R^2^ (0.9946) as shown in Table 3. It was found that on increasing the concentration LC (A), CS (B) and TPGS (C), the drug release increase because of TPGS and LC had emulsifying and solubilizing property, increased the solubility of AN and decreased the interfacial tension between drug and releases media, hence increasing the drug release as compared to pure AN. It is also due to the fact that when increasing the concentration of TPGS, it increases the solubility of CS at neutral pH and enhances the diffusion of AN from nano formulation [35].

### 3.5. Point Prediction

The hybrid AN-NPs were further optimized from the point prediction method. The optimized formulation (ON2) showed the practical particle size (Y_1_) of 192.6 ± 4.2 nm, encapsulation efficiency (Y_2_) 69.3 ± 1.1% and drug release (Y_3_) 61.51 ± 2.5%. The predicted value of the ON2 showed PS of 196.1 nm, EE of 68.1% and drug release of 59.7%. The low deviation in the results was observed between actual and predicted value of each response and revealed that the model was well fitted. The concentration of formulation factor (LC, CS and TPGS) was further varied and the observed responses depicted in Table 4.

### 3.6. Particle Size, PDI and Zeta Potential

**Figure 5:**
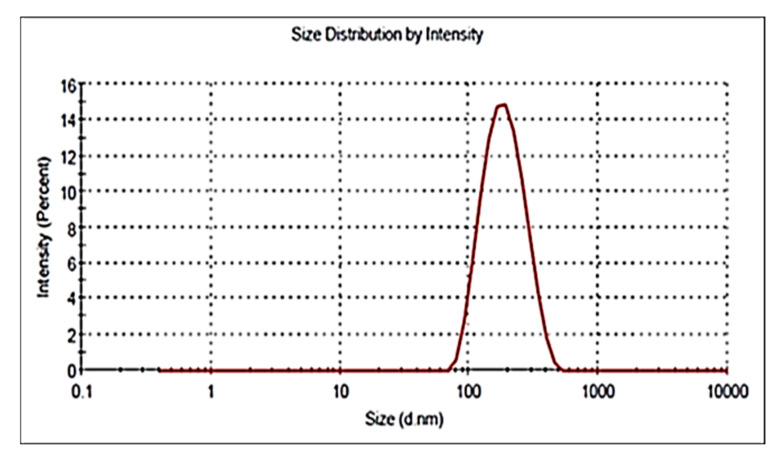
Particle size image of ON2.

**Figure 6:**
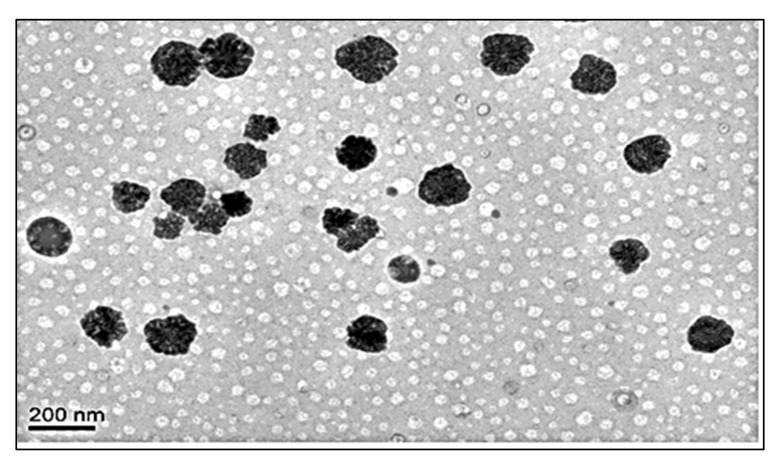
Surface morphology image of ON2.

The particle size, PDI and zeta potential of optimized formulation (ON2) was found to be 192.54 ± 4.23 nm (Figure 5), 0.26 and + 36.54 mV. The particle was found to be <200 nm and it is good for cancer cells. A size less than 200 nm could easily enter into cancer tissue due to the enhanced permeability and retention into tissue. The increased circulation time diminishes the opsonization and is identified by macrophages [36,37]. PDI is <0.5 indicates uniform size distribution and homogeneity. The high positive zeta potential of CS revealed that NPs were stable and disaggregated in form. The surface charge (ZP) on the vesicle is very important for cellular interaction and uptake, the value ± 30 mV is standard for stability [38]. The higher negative or positive value indicates greater stability. The prepared formulation showed the value within the standard range. The value depicted a characteristic property followed by flocculation exceeding repulsive force [39]. In this study, the surface has been completely coated with the cationic charge chitosan, and the repulsion among the NPs takes place [40]. The cationic charge easily binds with the negative-charged intestinal mucin and helps to improve the drug’s bioavailability and therapeutic activity [41]. The morphology of the optimized formulation (ON2) was analyzed and exhibited non-aggregated and spherical shape particles (Figure 6).

### 3.7. Fourier-Transform Infrared Spectroscopy (FTIR)

**Figure 7:**
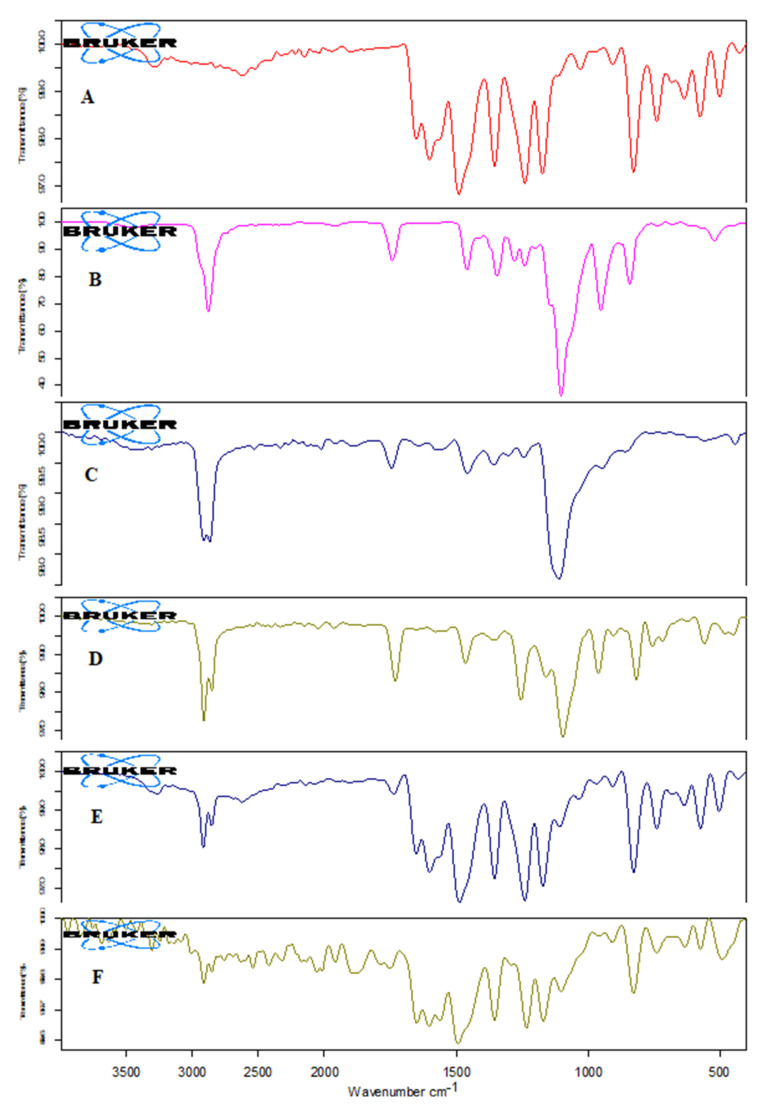
IR spectra of (**A**) Apigenin; (**B**) TPGS; (**C**) Chitosan; (**D**) Lecithin; (**E**) physical mixture; and (**F**) ON2.

The IR spectra of AN, TPGS, CS, LC, physical mixture, and formulation (ON2) were analyzed and the results are depicted in Figure 7. The AN showed the characteristic stretching vibration peaks of 3276.40 cm^−1^ (OH group of phenol), at 2614.44 cm^−1^ (CH starching vibration of CH_2_), 1651 cm^−1^ and 1602 cm^−1^ (C = O) and at 1130 cm^−1^ (due to C-O), respectively. These peaks confirm the purity as well as the structure of AN (Figure 7A). TPGS exhibited main identification characteristic peaks at 2879.25 cm^−1^ and 1742.25 cm^−1^ due to stretching vibrations of the aliphatic C-H and stretching of the C = O group confirming the purity of TPGS (Figure 7B). The spectra of CS showed the 3457.94 cm^−1^ stretching vibration due to the –NH_2_, and –OH functional group. However, peaks at 1642.39 cm^−1^ and 1572 cm^−1^ due to CONH_2_ and NH_2_ (bending vibration) confirmed the purity of CS (Figure 7C). The LC spectra showed the C-H stretching vibration peaks at 2910.07 cm^−1^, and 2852.08 cm^−1^, a stretching peak of the ester carbonyl group at 1730.75 cm^−1^ and after a C-O peak at 1257.20 cm^−1^ (Figure 7D). The physical mixture showed the peaks at 3267.07 cm^−1^ of OH, 2917.16 cm^−1^ of the OH group of phenol, C-H stretching at 2917.16, C = O stretching at 1733.28, and C-O stretching peaks at 1107.68 cm^−1^ (Figure 7E). In the spectra of optimized formulation (ON2) the drug peak’s intensity reduces and disappears (Figure 7F), confirming that the drug was encapsulated into the hybrid nanoparticle core.

### 3.8. Thermal Analysis

**Figure 8:**
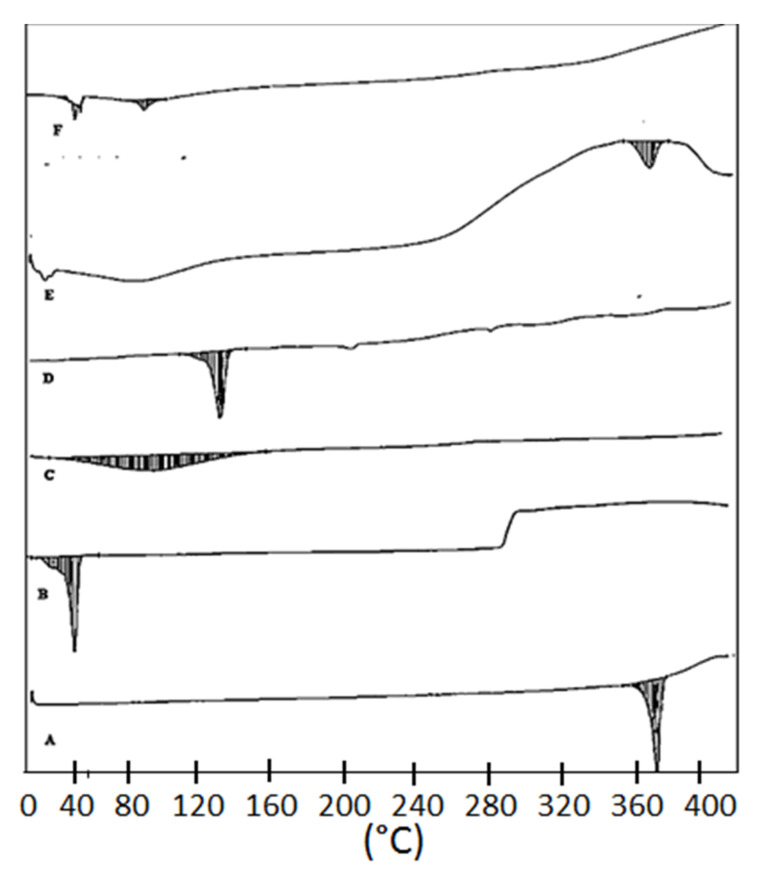
DSC thermogram of (**A**) Apigenin, (**B**) TPGS, (**C**) Chitosan, (**D**) Lecithin (**E**), physical mixture, (**F**) ON2.

The thermal spectra of AN, TPGS, CS, LC, the physical mixture, and the optimized formulation (ON2) were analyzed and the results are depicted in Figure 8. The spectra of AN has the characteristic endothermic sharp peaks at 361.55 °C assuring the purity and its crystalline nature (Figure 8A). TPGS, CS and LC exhibited peaks at 41.19 °C (Figure 8B), 87.82 °C (Figure 8C) and 122.97 °C (Figure 8D). The physical mixture exhibited an AN peak at 341.32 °C and it slightly shifted to a lower melting point (Figure 8E). The AN peak was absent in the formulation (ON2) thermogram. It may be due to the fact the drug was dissolved or encapsulated into lipid polymeric nanoparticle matrix (Figure 8F) which agreed with previous work.

### 3.9. In-Vitro Drug Release

**Figure 9:**
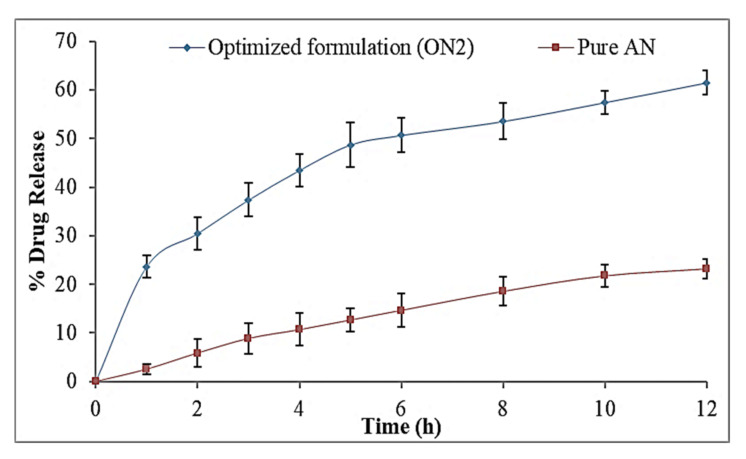
In vitro release study graph of pure Apigenin and optimized formulation (ON2). The study was performed in triplicate and data shown as mean ± SD.

The release study of the ON2 and the pure AN was performed by the pretreated dialysis bag method and results represented in Figure 9. The optimized formulation (ON2) showed the dual release pattern of AN, initially fast release (23.56 ± 2.2% in 1 h) and later sustained release was found up to 12 h (61.5 ± 2.5%). The initial fast release of AN is due to the release of the surface-deposited drug and later, the slow release due to the release of AN from inside the core matrix of the hybrid NPs. The ON2 exhibited significantly (*p* < 0.05) higher release than the pure AN. The high release was found due to the presence of solubilizing agent lecithin and the TPGS. The higher drug release was due to the nano metric size and the availability of a greater effective surface area. The effective surface area increases and that then gives an increase in the contact point of the drug to the dissolution medium [42]. The presence of CS in the NPs leads to sustained release which provides the extra boundary [43,44]. The reason for the slower drug release is that the drug is encapsulated into the inner core of NPs and the drug is released by the diffusion and erosion or the swelling of the carrier [45]. The TPGS provides the homogeneous mixing of AN with polymer and lipid because of its efficient emulsifying properties and direct effects on the release of drug from the formulation matrix [46]. The release data of ON2 were applied in release kinetic models and the Korsmeyer–Peppas model was found to be the best fit (R^2^ = 0.9829) which explained the sustained release pattern of the drug. The exponent ‘n’ value is 0.39 (<0.5) indicated the Fickian Diffusion transport pattern of the drug and it is a suitable method for effective delivery of the drug through the oral route.

### 3.10. Ex Vivo Permeation Study

**Figure 10:**
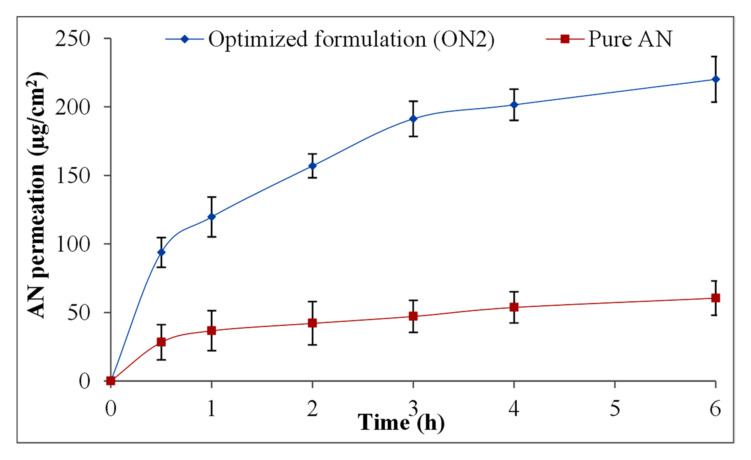
Ex vivo permeation study graph of pure Apigenin and optimized formulation (ON2). The study was performed in triplicate and data shown as mean ± SD.

The excised rat intestine was used for ex vivo permeation study of pure AN and optimized AN-LC-CS-TPGS-NPs (ON2) and the results expressed in Figure 10. The optimized AN-LC-CS-TPGS-NPs (ON2) exhibited remarkably high permeation (220.2 ± 16.6 µg/cm^2^) than pure AN (60.6 ± 4.6 µg/cm^2^) in 6 h of study. The flux value exhibited 3.9-fold (31.55 ± 4.1 µg/cm^2^.h) more than pure AN (8.11 ± 1.2 µg/cm^2^.h). The ON2 showed the APC of 32.87 × 10^−4^ cm/s which is 3.8-fold higher than the pure AN (12.94 × 10^−4^ cm/s). The low permeation of pure AN is due to the thickness and small porosity of the intestine and the high wide clearance of the intestinal P-gp efflux [47]. The significantly high permeation of the ON2 is due to the nano size and high surface area of formulation over the intestinal area [48]. The emulsifying property of LC may interact with mucus triggering mucin layer and little disorganized and finally encourage the intestinal permeation [49]. In addition, the interaction between positive charge CS and negative charge mucin layer of intestine lining could improve the paracellular transport of drugs by intestinal tissue [50]. The enhanced permeation is also due to the presence of TPGS, which may decrease the interfacial tension between formulation and intestinal tissue (epithelial layer) and increase the contact of the drug with tissue [51].

### 3.11. Antioxidant Activity

DPPH Method: The comparative antioxidant potential of the pure AN and the ON2 was performed by DPPH method. The data displayed that the antioxidant potential is directly proportional to the concentration of AN. On increasing the concentration of AN, the antioxidant potential was increased. A significantly (*p* < 0.05) higher activity was found in the ON2 than the pure AN at all concentrations. The ON2 exhibited maximum activity at 100 µg/mL concentration (96.2 ± 4.9%), however the pure AN showed 72.5 ± 5.1% at 100 µg/mL. The significant (*p* < 0.05) high activity of the AN in optimized hybrid nanoparticles (ON2) is due to the high solubility of the AN. The AN has potent antioxidants and robust scavengers of free radicals. The antioxidant activity was increased due to increased solubility of AN in-hybrid nanoparticle in presence of LC and TPGS.

ABTS scavenging method: The antioxidant activity of the pure AN and optimized hybrid nanoparticles (ON2) is also performed by ABTS method. The result displayed concentration-dependent activity between 10–100 µg/mL. There is a significant (*p* ˂ 0.05) difference in the activity was observed between pure AN and optimized hybrid nanoparticles (ON2). The maximum effect was observed at 100 µg/mL concentration. There was a non-significant difference in the activity was observed at 50 µg/mL (73.7 ± 4.1) and 100 µg/mL (79.8 ± 3.9) in the ON2. The activity was found to be similar to the DPPH activity. The standard BHT also showed antioxidant potential but its activity was closer to pure AN. The optimized hybrid nanoparticles (ON2) showed higher activity due to the enhanced solubility of AN after incorporation in NPs. The result was found to be similar to DPPH activity.

### 3.12. Cell Viability Study

**Figure 11:**
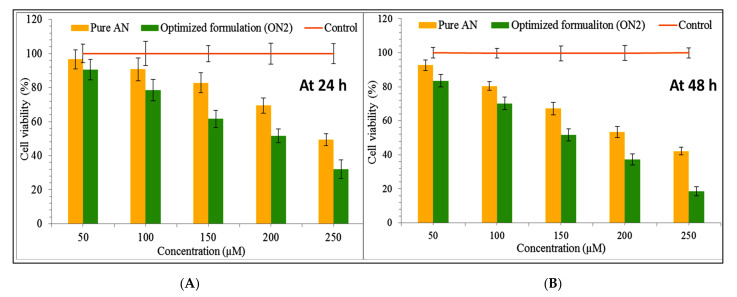
In vitro cell viability study graph of pure Apigenin and optimized formulation (ON2) treated with breast cancer cell line (**A**) 24 h of study; (**B**) 48 h of study. The study was performed in triplicate and data shown as mean ± SD.

Figure 11A,B showed the anticancer activity of the pure AN and the ON2 on the MCF 7 cell line in a concentration-dependent manner (50–250 µM). The results showed significant effects from the pure AN and the formulation (ON2) at all tested concentrations in comparison to the control. The maximum effect was found from 250 µM at 24 h and 48 h. There was a highly significant (*p* < 0.001) effect found between the pure AN and the ON2 at 250 µM concentration. The formulation (ON2) exhibited lesser cell viability (31.98 ± 3.23%) than pure AN (49.39 ± 4.23%) at 24 h of study. The pure AN showed 42.11 ± 3.21% cell viability, whereas ON2 showed 18.55 ± 2.76% viability at 48 h. There was slightly greater effect was achieved from pure AN at 48 h in comparison to 24 h result. The formulation (ON2) showed highly significant effect at 48 h (18.55 ± 2.76%) in compare to the 24 h result (31.98 ± 3.23%). Further, the IC_50_ value was calculated and the pure AN showed 248 µM and 221 µM at 24 h and 48 h of study, respectively. The formulation (ON2) exhibited IC_50_ value of 224.32 µM and 165.11 µM at 24 h and 48 h, respectively. The difference was found to be significant (*p* < 0.05). The lower IC_50_ value was found more from the ON2 than the pure AN. The lesser IC_50_ value is due to the nano size of the formulation and the high solubility of AN in the presence of lipids and TPGS. The AN showed this activity by inhibiting the estrogen-receptor signaling pathway in the MCF-7 cancer cell [52]. The cell line viability study reported by different researchers, reported marked activity of AN in different cell lines. Sen et al., formulated AN-PLGA nanoparticles and exhibited 7.8 and 10.8 times less IC_50_ than the pure drug against a lung cancer cell line (B16F10 and A549) [53]. In another study, Zhai et al., formulated and exhibited approximately 5-fold lesser IC_50_ value against HepG2 and MCF-7 cell lines than pure AN solution [45]. AN has been reported to reduce the tumor growth in a dose-dependent manner and acts by decreasing the cell proliferation in clonogenic cancer cells (45). The apoptosis associated with an increased level of Caspase 3 and Bax/Bcl-2 ratio led to cell death [1,54]. From the study, it can be concluded that the ON2 depicted significantly (*p* < 0.001) enhanced activity due to the greater AN solubility. As the solubility increases, the higher AN concentration reaches the target site. The use of chitosan also promotes the anticancer activity by enhancing the drug permeation through enhancing the muco-adhesion. It also helps to open the tight junction of the cell wall leading to an accumulation of the drug [55]. It also promotes apoptosis by activating procaspase, triggered from outside the cell to accelerate the cleavage and thus cell death [56].

### 3.13. Antimicrobial Study

The antibacterial activity of the pure AN and the optimized AN-LC-CS-TPGS-NPs (ON2) were found through the cup-plate method against *B. subtilis* and *S. typhimurium*. The ZOI of the pure AN against *B. subtilis* and *S. typhimurium* was found to be 10.25 ± 0.6 mm and 16.6 ± 1.1 mm, respectively. However, the optimized formulation AN-LC-CS-TPGS-NPs (ON2) exhibited ZOI against *B. subtilis* and *S. typhimurium* of 15.6 ± 0.8 mm and 20.7 ± 0.3 mm, respectively. The standard tetracycline solution was taken as standard and the zone of inhibition was found to be 20.9 ± 0.4 mm against *B. subtilis* and 22.5 ± 0.6 mm against *S. typhimurium.* The study result revealed that the optimized AN-LC-CS-TPGS-NPs (ON2) showed significant (*p* < 0.05) enhancement in antibacterial activity against both the microorganisms than pure AN. The ON2 showed enhanced activity than the pure AN due to its nano-metric size, enhanced solubility and permeability of the AN. The nano-sized particles gets a greater effective surface area to be available for absorption. The surfactant used in the preparation of NPs helps to enhance the solubility which promotes enhanced AN available to produce therapeutic efficacy. In addition, the chitosan used in ON2 also reported for antibacterial activity [57] Yilmaz et al., 2020). The chitosan showed the antibacterial activity via binding with the negatively charged bacterial cell wall causing disruption of the cells. It also helps to alter the membrane permeability, followed by inhibition of DNA replication to lead to cell death [58]. The AN acts by the mechanism of inhibiting the nucleic acid, cell wall synthesis/lysis of bacterial cell as well as inhibiting the DNA-gyrase [59,60]. Banerjee and associates developed the AN-loaded liposome and exhibited significant high activity than pure AN against *S. aureus*, *B. subtilis*, *P. aeruginosa* and *E. coli* [59].

## 4. Conclusions

APG-loaded hybrid nanoparticles were successfully prepared using lipid as well as chitosan. The formulation was optimized by Box–Behnken design using the different independent variables. The optimized formulation (ON2) was selected by the point prediction method. Finally, the antioxidant, antimicrobial and cytotoxicity activity was assessed to check the in vitro potential of prepared formulation. The formulation (ON2) exhibited particle sizes of 192.5 ± 4.3 nm, encapsulation efficiency of 69.35 ± 1.1% and drug release of 61.5 ± 2.5%. The PDI value depicted high homogeneity (<0.5) and the high positive zeta potential also supported the stability of formulation. The formulation (ON2) depicted a sustained drug release profile and also the high permeation profile justified the rationale of the study. The antioxidant and cytotoxicity study exhibited significant higher activity than pure AN due to the presence of LC and TPGS with CS. It helps to enhance the solubility of poorly soluble APG. Finally, the formulation exhibited significantly (*p* < 0.05) enhanced antimicrobial activity against Gram-positive and Gram-negative bacteria than pure AN.

## Figures and Tables

**Table 1:** Formulation composition of Apigenin-loaded hybrid nanoparticles and their effects on particle size, encapsulation efficiency and drug release.

Code	Formulation Variables			Responses					
	Lecithin (A) (mg)	Chitosan (B) (mg)	TPGS (C) (%)	Particle Size (nm)		Encapsulation Efficiency (%)		Drug Release (%)	
				Actual	Predicted	Actual	Predicted	Actual	Predicted
				Value	Value	Value	Value	Value	Value
F1	80	20	0.75	101.28	103.13	52.65	53.80	36.47	37.61
F2	180	20	0.75	171.32	170.53	63.12	62.61	60.40	63.41
F3	80	60	0.75	190.04	189.83	68.23	70.14	56.60	58.59
F4	180	60	0.75	227.24	225.39	79.54	77.95	53.53	55.39
F5	80	40	0.5	162.29	160.88	60.4	63.39	52.75	54.46
F6	180	40	0.5	201.21	204.43	71.04	70.19	62.51	60.35
F7	80	40	1	175.21	174.99	64.54	63.56	56.50	55.66
F8	180	40	1	245.00	244.40	71.34	73.36	67.11	65.39
F9	130	20	0.5	131.58	133.14	56.32	57.12	54.78	53.13
F10	130	60	0.5	204.75	203.37	74.12	73.46	58.63	56.93
F11	130	20	1	159.25	156.63	61.56	60.29	57.38	55.07
F12	130	60	1	230.52	231.96	77.13	76.63	66.19	64.04
F13 *	130	40	0.75	184.05	185.75	67.78	66.88	59.32	58.60
F14 *	130	40	0.75	187.43	185.75	67.23	66.88	58.45	58.60
F15 *	130	40	0.75	185.76	185.75	68.13	66.88	58.04	58.60

* center point.

**Table 2:** ANOVA of best fit model of independent variables (PS -Y_1_, encapsulation efficiency -Y_2_, drug release -Y_3_).

	Particle Size (PS)			Entrapment Efficiency			Drug Release		
Source	Sum of Squares	F Value	*p*-Value Prob > F	Sum of Squares	F Value	*p*-Value Prob > F	Sum of Squares	F Value	*p*-Value Prob > F
Model	19,436.62	1475.44	<0.0001	746.56	164.68	<0.0001	694.55	287.73	<0.0001
A-Lecithin	5829.03	3982.34	<0.0001	192.27	127.24	<0.0001	212.52	792.36	<0.0001
B-Chitosan	10,448.62	7138.41	<0.0001	534.15	353.48	<0.0001	83.94	312.98	<0.0001
C-TPGS	1516.55	1036.10	<0.0001	20.13	13.32	0.0038	42.79	159.54	<0.0001
AB	269.61	184.19	<0.0001	-	-	-	182.25	679.51	<0.0001
AC	238.16	162.71	<0.0001	-	-	-	0.18	0.67	0.4515
BC	0.90	0.61	0.4679	-	-	-	6.17	23.01	0.0049
A^2^	1.16	0.79	0.4127	-	-	-	37.56	140.05	<0.0001
B^2^	707.13	483.11	<0.0001	-	-	-	49.56	184.79	<0.0001
C^2^	341.51	233.32	<0.0001	-	-	-	68.35	254.85	<0.0001
Residual	7.3185			16.62	-	-	1.34		
Lack of Fit	1.60	0.18	0.8972	16.62	8.750732	0.1067	0.49	0.38	0.7814
Pure Error	5.712	-	-	0.41	-	-	0.85		
Cor Total	19,443.94	-	-	763.18	-	-	695.89		<0.0001

**Table 3:** Model statistical summary of all applied models for selected responses.

Particle Size						
Source	Std. Dev.	R^2^	Adjusted R^2^	Predicted R^2^	PRESS	
Linear	12.24	0.9151	0.8920	0.8191	3516.00	
2FI	11.94	0.9413	0.8973	0.6911	6004.99	
Quadratic	1.21	0.9996	0.9989	0.9980	38.55	Suggested
**Encapsulation Efficiency**						
Linear	1.22	0.9782	0.9722	0.9566	33.09	Suggested
2FI	1.19	0.9849	0.9735	0.9334	50.80	
Quadratic	0.98	0.9936	0.9823	0.9064	71.38	
**Drug Release**						
Linear	5.69	0.4875	0.3477	−0.0884	757.41	
2FI	4.58	0.7585	0.5774	−0.24752	868.13	
Quadratic	0.51	0.9980	0.9946	0.9861	9.70	Suggested

**Table 4:** Optimization by point prediction method and value at 95% PI low and 95% PI high.

LC (mg): CS (mg): TPGS (%)	Responses	Actual value	Predicted value	95% PI Low	95% PI High	% Error
135.45:43.75:0.77	Y_1_ (PS)	192.6 ± 4.2	196.1	192.51	199.66	−1.84
	Y_2_ (EE)	69.35 ± 1.1	69.1	66.28	71.88	0.40
	Y_3_ (DR)	61.51 ± 2.5	59.7	58.17	61.23	2.93

## Data Availability

Not applicable.
